# Brain blood flow and hyperventilation on cold water immersion: can treading water help control these symptoms of cold shock?

**DOI:** 10.1186/2046-7648-4-S1-A40

**Published:** 2015-09-14

**Authors:** Martin Barwood, Holly Burrows, Jess Cessford, Liz Fraser, Stuart Goodall, Scott Griffiths

**Affiliations:** 1 Dept. Sport, Exercise & Rehabilitation, Northumbria University, UK; 2 Royal Victoria Infirmary, Emergency Medicine Unit, Newcastle-Upon-Tyne, UK

## Introduction

Cold-water immersion (CWI) elicits the cold shock response (CSR). The hyperventilatory component of the CSR causes a decrease in cerebral blood flow velocity (CBFv) potentially causing sensations of dizziness and increasing the risk of becoming unconscious and consequently drowning [1]. In these early minutes of CWI the current advice is to 'float first' and remain stationary [2] yet this strategy may not have any effect on ventilation and therefore brain CBFv. We tested the hypothesis that leg only exercise could offset the reduction in CBFv in a resting CWI (H_1_) and be absent in warm water immersion.

## Methods

Seventeen participants consented and visited the laboratory 3 times; mean [SD]: age 21 [3]yrs; height 1.71 [.01]m; mass 70.9 [10.1]kg. All immersions were standardised by depth, duration, clothing (bathing suit) and time of day. Test conditions were a) a resting warm water immersion (WWI; 34.7 [2.6] °C), b) a resting CWI (CWI-R: 12.2 [0.5] °C), c) a CWI (12.1 [0.5] °C) where light exercise (leg kicking/treading water; 80 bpm^-1^) commenced 30-seconds after water entry (CWI-K). CBFv was measured using a transcranial Doppler at a fixed depth (61 [1] mm) over the middle cerebral artery. Oxygen uptake and ventilation were measured using an online gas analysis system. Perceptions of breathlessness were measured after 1, 3 and 5 minutes using an 11-point categorical scale (0-*not at all breathless*, 10-*extremely breathless*). ANOVA was used to analyse the data to an alpha level of 0.05.

## Results

CWI induced significant changes in contrast to WWI (see Table 1).

**Table 1:** Mean [SD] perceived breathlessness, CBFv, oxygen uptake, and carbon dioxide production in WWI (condition a), CWI-R (b) and CWI-K (c); letters denote differences between the corresponding condition.

	CBFv (Δ%)			VO_2 _(mL.kg^-1^.min^-1^)			VCO_2 _(mL.kg^-1^.min^-1^)		
	**WWI^a^**	**CWI-R^b^**	**CWI-K^c^**	**WWI^a^**	**CWI-R^b^**	**CWI-K^c^**	**WWI^a^**	**CWI-R^b^**	**CWI-K^c^**

PRE	-	-	-	387[96]	407[58]	405[90]	335[80]	377[75]	365[87]

1 MIN	5[4]^b^	-6[9]^a^	-3[16]	633[117]^c^	671[129]	692[137]^a^	518[97]^b,c^	837[253]^a^	880[343]^a^

2 MIN	3[6]^b^	-6[9]^a^	2[20]	424[84]^c^	437[94]^c^	534[89]^a,b^	375 [79]^c^	482[212]^c^	623[216]^ab^

3 MIN	3[4]	1[10]	3[16]	390[76]^bc^	432[84]^ac^	537[79]^a,b^	347 [76]^c^	405[173]^c^	497[133]^ab^

4 MIN	3[4]	7[11]	8 [21]	359[66]^bc^	436[101]^ac^	543[84]^a,b^	321[60]^c^	368[135]^c^	460[120]^ab^

5 MIN	5[6]	7[10]	4[17]	362[72]^bc^	454 [85]^ac^	570[99]^ab^	322[66]^c^	372[108]^c^	455[99]^ab^

## Discussion

Leg kicking on CWI partially offset the reduction in CBFv that normally occurs on CWI; in contrast to a warm water control. WWI CBFv was only different to the CWI-R condition. This did not alleviate symptoms of breathlessness despite increased oxygen uptake and carbon dioxide production in the CWI-K condition; the hypothesis is only partially supported.

## References

[B1] Mantoni T Rasmussen JH Belhage JH Pott FC Voluntary respiratory control and cerebral blood flow velocity upon ice-water immersion. Aviation Space and Environmental Medicine 2008 79 8 765 768 10.3357/ASEM.2216.2008 18717115

[B2] Barwood MJ Bates V Long GM Tipton MJ Int J Aq Res Edu 2011 5 147 163

